# Vagal maneuvers and pharmacological cardioversion in stable PSVT: A systematic review with implications for emergency nursing practice

**DOI:** 10.1016/j.isci.2026.117869

**Published:** 2026-10-22

**Authors:** M. González-García, L. Carrillo-Franco, C. Morales-Luque, I. Rocha, M.S. Dawid-Milner, M.V. López-González

**Affiliations:** 1 Department of Human Physiology, Faculty of Medicine, University of Malaga, Málaga, Spain; 2 IBIMA Plataforma BIONAND, Málaga, Spain; 3 Unit of Neurophysiology of the Autonomic Nervous System (CIMES), University of Malaga, Málaga, Spain; 4 Lisbon School of Medicine and CCUL@Rise, Universidade de Lisboa, 1649-028 Lisbon, Portugal

**Keywords:** supraventricular tachycardia, Valsalva maneuver, adenosine, non-pharmacologic cardioversion, emergency nursing, emergency care, systematic review

## Abstract

Paroxysmal supraventricular tachycardia (PSVT) is a common arrhythmic emergency for which vagal maneuvers offer a non-pharmacological route to cardioversion before drug escalation. This systematic review compared the effectiveness and safety of vagal maneuvers—particularly the standard and modified Valsalva maneuvers—against pharmacological cardioversion in adults with stable PSVT. Following PRISMA 2020 guidance, eleven studies published between 2014 and 2024 were identified across six databases and synthesized narratively owing to clinical and methodological heterogeneity. The modified Valsalva maneuver consistently achieved higher cardioversion rates than the standard technique and carotid sinus massage, with a favorable safety profile, while adenosine remained effective but more frequently associated with recurrence and discomfort. Effectiveness varied by age, arrhythmia subtype, and care setting. Collectively, the evidence supports the modified Valsalva maneuver as a safe, low-cost, first-line option before pharmacological escalation, with particular relevance to nurse-led management in emergency and prehospital care.

## Highlights

• Modified Valsalva outperforms standard Valsalva and carotid massage in stable PSVT
• Its safety profile is consistent across settings, with only mild adverse events
• Early modified Valsalva reduces the need for adenosine and other drugs
• A simple, nurse-led technique for early rhythm control in emergency care

## Teaser

Interventions; Nursing research

## Introduction

Paroxysmal supraventricular tachycardia (PSVT) is a common arrhythmic emergency, typically characterized by sudden-onset narrow QRS tachycardia caused by reentry circuits involving the atrioventricular (AV) node, such as AV nodal reentrant tachycardia (AVNRT).^1^^,^^2^ Its incidence is estimated at 35 per 100,000 persons annually, with a prevalence of 2.3 per 1,000 and a higher occurrence among women and older adults. Most episodes are hemodynamically stable and occur in settings where non-specialist or nursing-led care is first-line—such as prehospital or rural environments—underscoring the relevance of safe, effective, and resource-efficient strategies like vagal maneuvers.^3^^,^^4^

The standard Valsalva maneuver (sVM) is the most widely recommended non-pharmacological technique for rhythm control in stable PSVT. It aims to activate the parasympathetic system via baroreceptor stimulation, transiently inhibiting AV nodal conduction.^5^^,^^6^ However, real-world effectiveness remains modest—often below 20%—due to inconsistent technique, insufficient intrathoracic pressure, and variable patient cooperation.^7^^,^^8^^,^^9^ Emerging evidence suggests that repeated or suboptimal maneuvers may impair baroreflex responses or cause hemodynamic instability, underscoring the need for more standardized, practical approaches.^10^

The modified Valsalva maneuver (mVM), introduced in the REVERT trial, adds postural repositioning and passive leg elevation immediately after strain, enhancing venous return and vagal response.^11^ This simple modification more than doubles conversion rates compared to sVM.^11^^,^^12^^,^^13^ Subsequent systematic reviews, meta-analyses and pragmatic trials support the superiority of mVM over both sVM and carotid sinus massage (CSM), consolidating its role as the preferred vagal strategy in contemporary clinical guidelines.^14^^,^^15^^,^^16^^,^^17^

The neurophysiological basis of vagal maneuvers lies in transient baroreceptor-mediated parasympathetic activation, which slows AV nodal conduction and can terminate reentrant tachycardias.^5^^,^^6^ Its feasibility using simple tools—such as a 10 mL syringe for standardized pressure generation—makes it well-suited for nurse-led and out-of-hospital care.^7^^,^^18^ Recent studies have validated practical variations, such as using wider bore syringes or ensuring passive leg elevation, to enhance physiological efficacy without increasing adverse events.^13^^,^^18^ Moreover, new data suggest that vagal efficiency may be reduced in patients with baroreflex dysfunction, adding urgency to efforts to optimize technique and early intervention.^19^

Despite advances in vagal techniques, adenosine remains widely used when mVM fails or is not attempted. While effective (>85% conversion), it often causes distressing transient symptoms (e.g., chest pain and flushing)^4^^,^^9^ and calcium channel blockers (CCBs) carry risks like hypotension. These limitations are particularly relevant in vulnerable populations—such as pregnant women, pediatric, or patients in low-resource settings—where safer, nurse-administered alternatives are desirable.^4^^,^^20^ Therefore, there is renewed interest in optimizing mVM as a safer and more tolerable first-line approach.^1^^,^^2^

Although multiple trials and meta-analyses support mVM efficacy, few reviews have integrated the comparative performance of vagal and pharmacologic strategies specifically from a context-sensitive, nursing-oriented perspective focused on emergency and prehospital implementation. Specifically, there is a lack of synthesis focused on patient subgroups (e.g., pediatrics and pregnancy), technical variations, and the practical implications for nursing-led arrhythmia management.

This systematic review addresses this gap by evaluating non-pharmacologic versus pharmacologic cardioversion strategies in adult patients with stable PSVT. Through a synthesis of randomized controlled trials (RCTs) and systematic reviews conducted since the emergence of mVM protocols, we explore comparative effectiveness, safety, and feasibility—highlighting the role of mVM as a first-line, non-invasive option for emergency and nursing practice.

## Methods

### Design and objectives

This systematic review was conducted in accordance with the Preferred Reporting Items for Systematic Reviews and Meta-Analyses (PRISMA) 2020 guidelines and registered in PROSPERO: CRD420251032213. It aimed to compare the effectiveness and safety of vagal maneuvers—particularly the sVM and mVM techniques—and pharmacological cardioversion (mainly adenosine) in adults with stable PSVT. Secondary objectives included evaluating tolerability, adverse effects, contraindications, and the clinical applicability of these interventions in emergency and prehospital care.

The review followed a two-phase protocol: (1) a comprehensive literature search and selection of eligible studies, and (2) a critical appraisal of methodological quality using validated tools. Only studies meeting minimum quality thresholds were included in the synthesis.

#### Eligibility criteria and PICO framework

The eligibility criteria were defined *a priori*, based on the population, intervention, comparison, and outcome (PICO) model, to ensure focused clinically relevant study selection:

• Population (P): adults (≥18 years) diagnosed with stable PSVT, irrespective of sex, comorbidities, or care setting (emergency department or prehospital environment).
• Intervention (I): vagal maneuvers, including both sVM and mVM techniques, as non-pharmacological methods to achieve cardioversion.
• Comparison (C): pharmacological cardioversion, primarily with adenosine and, in some cases, with CCBs (e.g., verapamil).
• Outcome (O): return to sinus rhythm (successful cardioversion), tolerability, adverse effects, and contraindications.

Studies were included if they met all the following criteria:

• Published between December 2014 and December 2024, in English. The starting point was chosen to capture the post-REVERT period, when the mVM began to be systematically studied.
• Available as full-text, peer-reviewed articles.
• Study designs: RCTs or systematic reviews/meta-analyses.
• Comparative evaluation between vagal maneuvers and pharmacological treatment in stable PSVT.
• Only studies involving adult human participants were retained.

Exclusion criteria:

• Studies involving pediatric patients, pregnant women in the third trimester, or hemodynamically unstable patients.
• Studies focused on other arrhythmias (e.g., atrial fibrillation, atrial flutter, or sinus tachycardia).
• Case reports, editorials, narrative reviews, conference abstracts, or studies without outcome comparison between interventions.
• Articles with critically low methodological quality based on validated assessment tools.

One retrospective cohort study^21^ was included as an acknowledged deviation from the pre-specified eligibility criteria. This decision was based on three considerations: (1) its rigorous retrospective design with adequate sample size (*n* = 76), (2) the provision of unique comparative data on weight-based adenosine dosing not available in any included RCT, and (3) its direct relevance to the pharmacological component of the review question. This deviation is explicitly acknowledged as a limitation of the review.

### Information sources and search strategy

Six databases were searched between January and April 2025: PubMed/Medical Literature Analysis and Retrieval System Online (MEDLINE), Excerpta Medica dataBASE (EMBASE), Cochrane Library, Cumulative Index to Nursing and Allied Health Literature (CINAHL), ProQuest Nursing & Allied Health Database, and Scopus. The search strategy combined controlled vocabulary (Medical Subject Headings [MeSH]/Emtree) and free-text terms, including:

• “paroxysmal supraventricular tachycardia” OR “PSVT”
• “Valsalva maneuver” OR “vagal maneuvers” OR “modified Valsalva”
• “adenosine” OR “verapamil” OR “pharmacologic cardioversion”
• “sinus rhythm” OR “cardioversion success”

The complete search strategy used in each database, together with the number of documents retrieved and selected, is presented in Table 1. Results were limited to studies in adults, published in English between December 2014 and December 2024. Duplicates were removed using Mendeley. Two reviewers independently screened titles, abstracts, and full texts. Disagreements were resolved by discussion with a third reviewer. Additional sources were identified through backward citation tracking.

**Table 1:** Search terms and strategies used across six major biomedical databases, including Boolean operators and controlled vocabulary where applicable

Source	Search strategy	Documents retrieved	Documents selected
MEDLINE	(Valsalva maneuver) AND (paroxysmal supraventricular tachycardia OR tsvp OR psvt OR paroxysmal tachycardia) AND (adult)	22	10
	(adenosine AND (paroxysmal supraventricular tachycardia OR tsvp OR psvt OR paroxysmal tachycardia) AND adults)	43	7
PubMed	(“Valsalva maneuver”[MeSH]) AND “tachycardia, supraventricular”[MeSH] AND “adult”[MeSH]	16	1
	(adenosine AND “paroxysmal supraventricular tachycardia” AND adult)	13	1
CINAHL	(Valsalva maneuver) AND (paroxysmal supraventricular tachycardia OR tsvp OR psvt OR paroxysmal tachycardia) AND (adult)	16	3
	(adenosine AND (paroxysmal supraventricular tachycardia OR tsvp OR psvt OR paroxysmal tachycardia) AND adults)	31	1
EMBASE	(Valsalva maneuver/exp OR “Valsalva maneuver”) AND (“paroxysmal supraventricular tachycardia”/exp OR “paroxysmal supraventricular tachycardia”) AND (“adult”/exp OR adult) AND [2014-2024]/py	22	4
	(adenosine/exp OR adenosine) AND (“paroxysmal supraventricular tachycardia”/exp OR “paroxysmal supraventricular tachycardia”) AND (“adult”/exp OR “adult”)	101	3
ProQuest Nursing & Allied Health DB	(Valsalva maneuver) AND (paroxysmal supraventricular tachycardia) AND (adult)	71	3
	(adenosine AND (paroxysmal supraventricular tachycardia) AND adult)	122	1
Scopus	ALL (paroxysmal AND supraventricular AND tachycardia OR tsvp OR psvt AND adult) TITLE (Valsalva AND maneuver) AND PUBYEAR > 2013 AND PUBYEAR < 2025	24	7
	TITLE-ABS-KEY (paroxysmal AND supraventricular AND tachycardia) AND TITLE (adenosine) AND PUBYEAR > 2013 AND PUBYEAR < 2025	17	2
Cochrane Library	(Valsalva maneuver) AND (paroxysmal supraventricular tachycardia) AND (adult)	6	0
	(adenosine AND (paroxysmal supraventricular tachycardia OR tsvp OR psvt OR paroxysmal tachycardia) AND adults)	17	0

Document counts indicate the number of records retrieved per strategy prior to duplicate removal. Note: The search period was restricted to articles published between December 2014 and December 2024.

### Critical appraisal and risk of bias assessment

To ensure methodological rigor, all included studies were subjected to structured critical appraisal using validated tools selected according to study design. For RCTs and the retrospective cohort study, the Critical Appraisal Skills Program Español (CASPe) checklist was used. This tool comprises 11 items; studies were considered of acceptable quality if they met a minimum of 7 items (≥64%), with no critical domains unmet. For systematic reviews and meta-analyses, the Measurement Tool to Assess systematic Reviews, version 2 (AMSTAR-II) instrument was applied, comprising 16 items. Studies rated as “critically low” quality (defined as more than one critical domain unmet) were excluded from the synthesis. The quality ratings obtained for each study are presented in Table 2 and were used to contextualize the weight assigned to individual findings during narrative synthesis—specifically, studies with more unmet items were discussed with greater caution and their conclusions treated as exploratory rather than confirmatory.

**Table 2:** Methodological quality assessment of included studies using the CASPe checklist (for RCTs and observational studies) and AMSTAR-II tool (for systematic reviews and meta-analyses)

Author (year)	Type	1	2	3	4	5	6	7	8	9	10	11	12	13	14	15	16	Tool
Çorbacıoğlu et al.^22^	RCT	+	+	+	+	+	−	+	+	+	+	+	–	–	–	–	–	CASPe
Ceylan et al.^14^	RCT	+	−	+	+	+	−	+	−	+	+	+	–	–	–	–	–	CASPe
Appelboam et al.^11^	RCT	+	+	−	+	+	+	+	+	+	+	+	–	–	–	–	–	CASPe
Daengbubpha et al.^23^	RCT	+	+	+	+	+	+	+	−	+	+	+	–	–	–	–	–	CASPe
Deck et al.^21^	Cohort	+	+	+	+	−	+	+	+	+	+	+	–	–	–	–	–	CASPe
Lu et al.^15^	Meta-analysis	+	+	+	+	+	+	+	+	+	−	+	+	+	+	+	+	AMSTAR-II
Shoukat et al.^24^	RCT	+	−	+	−	+	−	+	−	+	+	+	–	–	–	–	–	CASPe
Wang et al.^25^	RCT	+	+	+	+	+	+	+	−	+	+	+	–	–	–	–	–	CASPe
Pachchipulusu et al.^20^	Systematic Review	+	−	+	+	−	−	+	+	+	+	+	+	−	−	+	+	AMSTAR-II
Xiao et al.^26^	RCT	+	+	+	+	+	+	+	+	+	+	+	–	–	–	–	–	CASPe
Chen et al.^12^	RCT	+	+	+	+	+	−	+	+	+	+	+	–	–	–	–	–	CASPe

Each “+” indicates that the criterion was met or partially met; each “−” indicates that it was not met. Only studies meeting minimum quality thresholds were retained for synthesis. Note: Columns 12 to 16 are shaded in light gray to indicate that they correspond exclusively to AMSTAR-II criteria and apply only to systematic reviews and meta-analyses. For randomized controlled trials and the cohort study, these columns are therefore not applicable.

### Data extraction and synthesis

A standardized extraction form was used by two independent reviewers to record:

• Author, year, country
• Study design and sample size
• Patient characteristics and care setting
• Type of intervention and comparator
• Outcomes: cardioversion rate, recurrence, adverse effects
• Key conclusions

Disagreements were resolved by a third reviewer. Due to clinical and methodological heterogeneity—particularly in intervention protocols and outcome measures—a meta-analysis was not appropriate. A narrative synthesis was performed, organized around four core themes:

1 Effectiveness of sVMs vs. mVMs
2 Comparative efficacy of vagal vs. pharmacologic strategies
3 Safety and tolerability of the interventions
4 Real-world applicability in emergency and prehospital settings

Only explicitly reported outcomes were analyzed. No imputation of missing data was made, and no authors were contacted for clarification.

## Results

### Overview of study selection and characteristics

A total of 525 records were identified: 521 through electronic databases and 4 via manual screening. After removing 145 duplicates, 380 titles and abstracts were reviewed. Of these, 43 full-text articles were assessed, with 34 excluded (see PRISMA flow diagram, Figure 1). Eight full-texts could not be retrieved—an acknowledged limitation. Ultimately, 11 studies were included: 7 RCTs, 1 retrospective cohort,^21^ and 3 systematic reviews or meta-analyses. Of these, 9 were identified through database searches (43 full texts assessed − 34 excluded) and 2 were incorporated following re-evaluation of records identified through citation searching, as shown in the PRISMA flow diagram (Figure 1).

**Figure 1:**
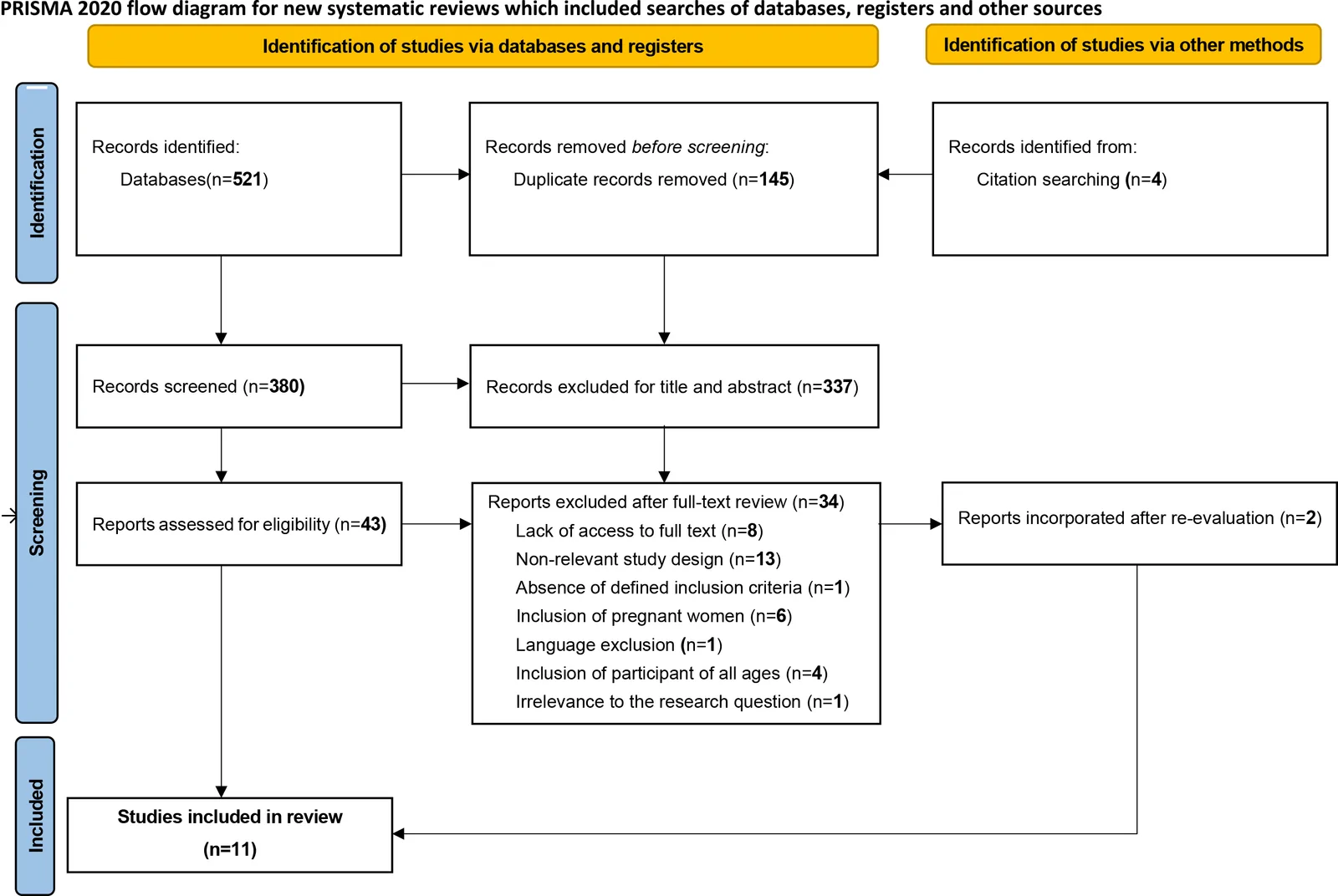
PRISMA 2020 flow diagram for new systematic reviews which included searches of databases, registers, and other sources

Study characteristics are summarized in Table 2. Most trials involved adult patients with hemodynamically stable PSVT, comparing sVMs and mVMs. Several also included pharmacological strategies such as adenosine or CCBs (see Table 3).

**Table 3:** Summary of included studies: Design, interventions, outcomes, and safety data

Author (year)	Design	Interventions	*N*	Care setting	PSVT subtype	1st attempt success (%)	Overall success (%)	Rescue drug use (%)	Adverse events	Primary outcome	Key findings
Çorbacıoğlu et al.^22^	RCT	sVM vs. mVM	56	emergency department	AVNRT/AVRT	sVM: 10.7% (3/28) mVM: 42.9% (12/28)	same as 1st (1 attempt only)	sVM: 89.3% (25/28) mVM: 57.1% (16/28)	mild dizziness in both groups (7.1%)	comparison of initial efficacy between maneuvers	significantly better success with mVM
Ceylan et al.^14^	RCT	sVM vs. mVM vs. CSM	98	emergency department	SVT (not specified)	mVM: 43.7% (14/32) sVM: 24.2% (8/33) CSM: 9.1%	mVM: 28.1% (9/32) sVM: 6.1% (2/33) (at 5 min)	not specified	not reported	immediate and 5 min cardioversion rates	mVM consistently outperforms others
Appelboam et al.^11^	multicenter RCT	sVM vs. mVM	428	emergency department (multicenter)	SVT (excl. AF/flutter)	sVM: 17% (37/214) mVM: 43% (93/214)	same as 1st (primary outcome at 1 min)	sVM: 69% (148/214) mVM: 50% (108/214)	mild, 5 cases of ventricular escape rhythms in mVM group	conversion to sinus rhythm	mVM highly effective and widely validated
Shoukat et al.^24^	RCT	sVM vs. mVM	62	emergency department	PSVT (not specified)	sVM: 6.5% (2/31) mVM: 22.6% (7/31)	not reported	sVM: 93.5% (29/31) mVM: 77.4% (24/31)	not reported	conversion after 1 attempt	low success rates, highlights variability
Wang et al.^25^	RCT	sVM vs. mVM	361	cardiology dept. (inpatient)	PSVT (not specified)	sVM: 15.4% (28/181) mVM: 47.8% (86/180)	sVM: 19.8% (36/181) mVM: 62.2% (112/180)	not specified	mild and self-limited	conversion rates at multiple attempts	mVM has clear clinical advantage
Chen et al.^12^	multicenter RCT	sVM vs. mVM	238	emergency department (multicenter)	AVNRT/AVRT	sVM: 16% (19/119) mVM: 46% (55/119)	sVM: 16% (19/119)	sVM: 71% (84/119) mVM: 45% (54/119)	minor, self-limited	effectiveness of mVM in emergency	mVM reduces need for drugs, effective early
		–	–		–		mVM: 46%				
Xiao et al.^26^	multicenter RCT	mVM vs. adenosine vs. combo	118	emergency department (multicenter)	AVNRT/AVRT	mVM: 24% (9/38) A: 30% (13/44) combo: 50% (18/36)	mVM: 42% (16/38) A: 75% (33/44) combo:	N/A	minor, transient	effectiveness of combined protocol	hybrid approach may be most effective
Lu et al.^15^	systematic review and meta-analysis	mVM vs. sVM	2527	various (meta-analysis)	PSVT (various)	RR: 2.05 (95% CI: 1.74–2.41)	RR: 1.80 (95% CI: 1.61–2.01)	less adenosine needed in mVM	RR 1.07; no significant difference	pooled success and adverse events	strong support for mVM superiority
Daengbubpha et al.^23^	pilot RCT	adenosine ± arm elevation	30	emergency department	PSVT (not specified)	standard: 80% (12/15) arm elevation: 86.7% (13/15)	same (first dose only)	all received adenosine	6.7% mild effects both groups	impact of posture effect	no significant difference, small N
Deck et al.^21^	retrospective cohort	<0.1 vs. ≥0.1 mg/kg adenosine	76	emergency department	PSVT (AVNRT/AVRT confirmed)	<0.1: 59.6% (31/52) ≥0.1: 92.7% (22/24)	same (first dose only)	all received adenosine	mild, no group differences	dose-related response to adenosine	supports weight-based dosing strategy
Pachchipulusu et al.^20^	systematic review	adenosine vs. verapamil	14 studies	various (systematic review)	PSVT (all types)	A: 81.8% V: 74.4% (Ballo 2004, *n*=106)	depends on HR	comparative	more frequent in A but tolerable	comparison of antiarrhythmic efficacy	heart rate influences drug choice

RCT, randomized controlled trial; mVM, modified Valsalva maneuver; sVM, standard Valsalva maneuver; SR, sinus rhythm; AVNRT, atrioventricular nodal reentrant tachycardia; AVRT, atrioventricular reentrant tachycardia; AF, atrial fibrillation; SVT, supraventricular tachycardia; ED, emergency department.

Percentages refer to the proportion of participants within each group achieving the specified outcome or experiencing adverse events. “rescue drug use” indicates the proportion of patients requiring pharmacological treatment (e.g., adenosine) following failure of vagal maneuvers.

### Methodological appraisal

Risk of bias was assessed using the CASPe checklist for RCTs and the AMSTAR-II tool for reviews. Appraisal results are detailed in Table 2 and discussed narratively due to design heterogeneity. A Grading of Recommendations, Assessment, Development and Evaluation (GRADE)-based certainty rating was not applied, in line with the narrative scope of this review.

The pooled sample across primary studies totaled 2,071 patients. Owing to the diversity of designs and outcomes, findings were synthesized narratively and structured by intervention type (Valsalva maneuver or pharmacological strategy) and outcome domain (efficacy, safety, and tolerability).

### Thematic synthesis of clinical findings

Findings were organized narratively by intervention type—non-pharmacological (Valsalva maneuvers) and pharmacological (adenosine)—and by outcome domains: efficacy, safety, and patient acceptability. This structure enables comparison across heterogeneous study designs.

#### Valsalva maneuvers: sVM vs. mVM

(1) Immediate efficacy in sinus rhythm restoration: Ceylan et al.^14^ directly compared sVM, mVM, and CSM with continuous electrocardiogram (ECG) monitoring. Immediate conversion rates were 43.7% (mVM), 24.2% (sVM), and 9.1% (CSM). Only the difference between mVM and CSM reached statistical significance (*p* = 0.0016), while comparisons between mVM and sVM (*p* = 0.0992) and between sVM and CSM (*p* = 0.1012) were not significant. Chen et al.^12^ using a structured mVM with supine positioning in the emergency department, also documented rapid rhythm restoration, although this was not their primary endpoint.
(2) Efficacy at 1 min after maneuver: 1-min cardioversion is a clinically relevant endpoint in emergency settings. Across included RCTs, mVM consistently showed higher success rates than sVM.

Çorbacıoğlu et al.^22^ reported 42.9% cardioversion with mVM versus 10.7% with sVM (*p* = 0.007). In a large UK trial, Appelboam et al.^11^ found mVM successful in 43% of cases after one attempt versus 17% with sVM (odds ratio [OR] 3.7, 95% confidence interval [CI]: 2.3–5.8; *p* < 0.0001). Wang et al.^25^ showed 47.8% success with mVM versus 15.4% with sVM, increasing to 62.2% and 19.8%, respectively, after up to three attempts (*p* < 0.001).

Ceylan et al.^14^ also reported 1-min success rates of 37.5% (mVM), 12% (sVM), and 6.1% (CSM), mirroring their immediate results. Shoukat et al.^24^ observed lower absolute rates (22.6% for mVM vs. 6.5% for sVM) with no statistically significant difference, possibly due to protocol variability or sample characteristics.

Lu et al.^15^ conducted a pooled analysis showing a risk ratio (RR) of 2.05 (95% CI: 1.74–2.41) for first-attempt success with mVM, and an overall RR of 1.80 (95% CI: 1.61–2.01; *p* < 0.001), confirming its superiority. Moderate heterogeneity (I^2^ = 66%) was noted, reflecting differences in implementation and study design without altering the overall trend.

Chen et al.^12^ using a mVM in emergency department, reported 46% overall cardioversion success (55/119 patients) after up to three Valsalva attempts.

(3) Efficacy at 5 min post-maneuver: although few studies assessed outcomes beyond 1 min, available evidence supports sustained efficacy of mVM. Ceylan et al.^14^ found 5-min conversion in 28.1% (mVM) versus 6.1% (sVM) and 3% (CSM), with significance for mVM vs. CSM (*p* = 0.0186) and a trend favoring mVM over sVM (*p* = 0.0926), despite limited sample size.

Wang et al.^25^ using a three-attempt mVM protocol, observed cumulative cardioversion rates up to 62.2%, indicating durable response over several minutes. Appelboam et al.^11^ similarly noted that most successful conversions occurred after the first mVM and were maintained during follow-up. Lu et al.^15^ included trials with delayed outcome assessments, confirming that mVM benefits extend beyond the immediate post-maneuver window—up to 10 min in some cases.

Chen et al.^12^ reported that most restorations occurred within 3 min of initiating mVM, supporting early but sustained effectiveness. Other trials^22^^,^^24^ did not evaluate conversion beyond 1 min, limiting mid-term conclusions.

(4) Adverse effects: across all 11 studies, the mVM showed an excellent safety profile, with only mild, transient adverse events and no serious complications reported.

Appelboam et al.^11^ noted minor events in 6% of mVM cases vs. 4% for sVM (*p* = 0.32), including brief idioventricular rhythms and self-limited asystole. Çorbacıoğlu et al.^22^ reported non-severe symptoms (dizziness and chest discomfort) in 7.1% of both groups. Wang et al.^25^ found adverse events in 11% (mVM) vs. 7.7% (sVM), with no clinical significance.

Lu et al.^15^ confirmed no significant difference in adverse events (RR = 1.07; 95% CI: 0.82–1.38) in a meta-analysis of 17 trials, most commonly reporting flushing, palpitations, and headache. Chen et al.^12^ described rare dizziness and abdominal discomfort after prehospital mVM.

Xiao et al.^26^ evaluating a hybrid protocol combining mVM with low-dose adenosine—a distinct intervention from mVM alone—found no arrhythmias and only minor flushing and chest pressure. Results from this study should not be generalized to the standalone use of mVM. In contrast, Deck et al.^21^ and Daengbubpha et al.^23^ reported higher adverse event rates (up to 8.3%) with pharmacologic strategies, primarily chest pain, and palpitations.

(5) Use of rescue pharmacological treatment following vagal maneuvers: several studies evaluated the need for pharmacological intervention after vagal maneuvers. Çorbacıoğlu et al.^22^ reported that 57.1% of mVM patients required drug therapy, compared to 89.3% in the sVM group (*p* = 0.007). Appelboam et al.^11^ found that adenosine was administered in 15% of mVM cases versus 45% in the sVM arm (treatment effect = 0.45; 95% CI: 0.30–0.68; *p* = 0.0002).

Shoukat et al.^24^ observed a high overall rescue rate (85.5%) using adenosine, verapamil, or metoprolol, though mVM showed a modestly higher pre-drug success (11.3% vs. 3.2%). Chen et al.^12^ reported a 12.9% adenosine use rate in a structured prehospital mVM protocol, notably lower than the 30.4% rate for sVM reported by Çorbacıoğlu et al. Xiao et al.^26^ focused on minimizing drug reliance via combined mVM and low-dose adenosine protocols, though specific rescue rates were not detailed. Lu et al.^15^ highlighted this trend but did not provide pooled estimates.

(6) Patient tolerance and acceptability: although tolerance and acceptability were not systematically evaluated across all studies, the available evidence suggests a favorable profile for mVM.

Wang et al.^25^ using a five-point Likert scale, found similar comfort ratings for mVM (3.67 ± 0.69) and sVM (3.54 ± 0.66; *p* = 0.064). Appelboam et al.^11^ described high patient acceptance and no increased discomfort from the postural component. Lu et al.^15^ cited minimal symptoms such as dizziness or pressure, with no significant difference between mVM and sVM.

Chen et al.^12^ noted high adherence and protocol completion in the emergency department. Xiao et al.^26^ reported no increase in discomfort with mVM-adenosine protocols, with only brief flushing or chest pressure.

#### Pharmacological cardioversion with adenosine: Effectiveness and comparison

Adenosine remains a key pharmacologic option for stable PSVT, particularly when vagal maneuvers are unsuccessful or not feasible. Included studies offer insights into its effectiveness, optimal administration techniques, and tolerability profile—informing both rescue use and hybrid strategies.

(1) Effectiveness of adenosine in PSVT reversion: adenosine’s efficacy varies with dose, delivery, and patient characteristics. In a prospective RCT, Daengbubpha et al.^23^ found comparable success using standard versus right-arm elevation methods (86.7% vs. 80%). Deck et al.^21^ observed significantly higher conversion with doses ≥0.1 mg/kg (92.7%) compared to lower doses (59.6%, *p* = 0.006).

Pachchipulusu et al.^20^ reported similar efficacy for adenosine (81.8%) and verapamil (74.4%), but noted that adenosine was less effective at heart rates <166 beats per min (bpm), whereas verapamil was less reliable above 186 bpm. Xiao et al.^26^ evaluated a hybrid protocol combining mVM and low-dose adenosine, achieving 86.1% success—higher than either mVM (42.1%) or adenosine alone (75%).

(2) Adverse effects and tolerability: adenosine is associated with transient symptoms—flushing, dyspnea, and chest pressure—which, though benign, may affect patient experience. Daengbubpha et al.^23^ reported minor side effects (dizziness and nausea) in two patients. Deck et al.^21^ found adverse events in 8.3% of higher dose cases, including transient arrhythmias and discomfort; the difference vs. lower doses (3.9%) was not statistically significant (*p* = 0.75).

Xiao et al.^26^ observed fewer symptoms in the mVM+adenosine group compared to adenosine alone, suggesting enhanced tolerability with combination protocols.

(3) Summary of evidence certainty: although no formal GRADE assessment was performed, convergence across trials and moderate-to-high methodological quality support the robustness of findings. Adenosine remains effective as a second-line option, but mVM—alone or in hybrid strategies—may offer improved tolerability and broader applicability, particularly in prehospital or resource-limited settings.

## Discussion

### General effectiveness and clinical applicability of vagal maneuvers

The synthesis of the eleven included studies confirms that vagal maneuvers—particularly the mVM—are effective, safe, and feasible first-line strategies for managing hemodynamically stable PSVT. The mVM consistently outperforms other non-pharmacological options in terms of both success rates and applicability across diverse clinical contexts.

The REVERT trial^11^ was pivotal in demonstrating that combining strain with supine repositioning and leg elevation nearly tripled the success rate compared to the standard technique. This low-cost, replicable protocol has since become a benchmark in both emergency and prehospital care.

Recent meta-analytic evidence reinforces these findings. Lu et al.^15^ confirmed superior efficacy of mVM without increased adverse events, while Huang et al.^27^ ranked it highest among vagal strategies (Surface Under the Cumulative Ranking curve (SUCRA) 1.0000 for initial success; 0.9992 for rhythm maintenance), with high-certainty evidence in key comparisons.

Real-world data also support its effectiveness. Ceylan et al.^14^ and Xiao et al.^26^ reported mVM superiority over standard techniques and CSM, with the latter study highlighting further gains from enhanced variants incorporating facial cold stimulation.

Feasibility in non-hospital settings is well established. Paramedic-led implementation has been effective,^28^^,^^29^ and early success correlates with reduced need for pharmacological rescue.^25^ Technical adaptations for specific patient populations further demonstrate the maneuver’s clinical flexibility.

In summary, robust evidence from RCTs, meta-analyses, and pragmatic studies consistently positions mVM as the most effective and operationally adaptable non-pharmacologic technique for initial PSVT management. Early use is especially advantageous where drug-free, low-resource interventions are preferred.

### Comparison with CSM: Efficacy and safety

The evidence consistently shows that CSM is less effective than Valsalva-based techniques, especially the mVM, for cardioversion in stable PSVT. In Ceylan et al.^14^ and Çorbacıoğlu et al.^22^ CSM yielded markedly lower success rates (≤9%) compared to mVM (≥37%), with significant differences in favor of mVM. The meta-analysis by Alfehaid et al.^30^ confirmed these findings, reporting lower pooled success rates for CSM and subgroup analyses with RRs clearly favoring mVM. These results reinforce that CSM is no longer a competitive first-line option.

Safety further limits CSM’s role. Though serious adverse events are rare, risks such as bradycardia or cerebral hypoperfusion are greater in older patients or those with carotid disease. Alfehaid et al.^30^ recommend caution in such populations. In contrast, large trials like REVERT^11^ and field studies^28^^,^^29^ reported no serious events with mVM, even in low-resource or prehospital environments.

From a procedural standpoint, mVM is easier to teach and apply. CSM requires precise anatomical knowledge and clinician skill, making it less viable in emergency or out-of-hospital contexts. Standardized mVM protocols, such as REVERT, are more practical and better suited for nursing or paramedic implementation.

The convergence of lower efficacy, safety concerns in vascularly compromised patients, and greater procedural skill requirements collectively argues against CSM as a first-line option in most emergency and nursing-led contexts. mVM should be prioritized not merely because it performs better, but because it performs better in precisely the settings where CSM carries the highest procedural and clinical risk.

### sVM vs. mVM: Comparative efficacy

A key finding of this review is the consistently greater effectiveness of the mVM over the sVM for cardioversion in stable PSVT. The REVERT trial^11^ remains the pivotal study, demonstrating a 43% success rate with mVM versus 17% with sVM (OR 3.7; 95% CI: 2.3–5.8; *p* < 0.0001) after a single attempt. An OR of 3.7 represents a clinically substantial effect—one that exceeds typical between-intervention differences seen in acute cardiac interventions—suggesting the postural modification constitutes a physiologically meaningful rather than merely incremental improvement. Its simple protocol—strain followed by supine repositioning and leg elevation—has since become the standard non-invasive approach in emergency settings.

Lu et al.^15^ reinforced these findings in a meta-analysis, reporting a RR of 2.05 (95% CI: 1.74–2.41) for first-attempt success and an overall RR of 1.80 (95% CI: 1.61–2.01), confirming consistent superiority without increased adverse events.

Smaller studies^14^^,^^22^ supported these results, with mVM showing higher success rates at 1 and 5 min post-intervention. Despite limited sample sizes, their findings aligned with those of larger trials.

The maneuver’s adaptability has been validated in diverse contexts. Prehospital and pediatric applications are addressed in dedicated sections of this review. Xiao et al.^26^ further demonstrated that the core protocol can be meaningfully augmented: a variant incorporating facial cold stimulation and low-dose adenosine achieved enhanced efficacy without added discomfort, suggesting the mVM framework is open to additive physiological enhancement.

The consistent superiority of mVM across trials of varied design, population, and clinical setting suggests an effect unlikely attributable to study-specific factors. The mechanistic basis—enhanced venous return during the Valsalva relaxation phase—provides physiological plausibility that strengthens confidence in the empirical findings beyond what statistical consistency alone would justify.

### Subgroup analyses: Type of reentry, age, and prehospital contexts

The effectiveness of mVM varies by arrhythmic mechanism, age, and care setting. Chen et al.^12^ found higher success in patients with short RP intervals (70%)—typically linked to AVNRT—versus 20% in long RP cases, supporting ECG-based stratification. This differential response has direct clinical implications: ECG-based arrhythmia subtype identification prior to mVM application may predict likelihood of success, supporting a stratified approach in which mVM is prioritized for confirmed AV-node-dependent arrhythmias while earlier pharmacological escalation is considered in long-RP patterns.

Age influences outcomes. Abbasi et al.^31^ showed lower efficacy of vagal maneuvers in children (∼47% with standard techniques), though modified approaches remain viable. Rayburn and Wagers^32^ reported successful pediatric cardioversion using a straw-assisted mVM, demonstrating its adaptability.

Prehospital implementation has proven feasible. Appelboam et al.^29^ and Gangaram et al.^28^ showed that trained paramedics can apply mVM effectively in ambulances. The evaluation of pre-hospital use of a Valsalva assist device in the emergency treatment of supraventricular tachycardia (EVADE) trial^25^ reported a 24% success rate with a Valsalva assist device, and early conversion reduced pharmacologic use and care costs.

These subgroup findings are clinically relevant not only for individual patient management but for institutional protocol design: a single standardized mVM protocol may be adequate across most adult emergency department presentations, but a modular approach—incorporating ECG-guided arrhythmia subtype identification, age-appropriate technical adaptations, and prehospital logistics—is likely to maximize population-level effectiveness.

### Pharmacologic alternatives and hybrid approaches

Though mVM is supported as a first-line approach for stable PSVT, pharmacological cardioversion remains an essential component of the treatment pathway when vagal maneuvers fail or are contraindicated. It is important to note that no included study directly compared mVM against adenosine or CCBs in a head-to-head randomized design; the comparative inferences drawn in this review are therefore indirect, derived from separate evidence streams. Adenosine achieves cardioversion in over 80% of cases^20^^,^^21^ but is associated with significant transient adverse effects—including chest pain, flushing, and dyspnea—that, while self-limiting, may impair patient experience and acceptability. Verapamil demonstrates comparable efficacy with a somewhat more favourable tolerability profile at lower heart rates, though its use requires greater caution in patients with hemodynamic borderline stability. The choice between agents should therefore be guided not only by efficacy but by heart rate, patient tolerance, available access, and setting—factors that are not yet systematically addressed in the included literature.

However, limitations exist. Ahmad et al.^33^ reported similar efficacy between adenosine and CCBs like verapamil, with better tolerability for verapamil in some cases. Pachchipulusu et al.^20^ found adenosine more effective at higher heart rates (>166 bpm), whereas verapamil performed better at lower rates—supporting rhythm-specific drug selection.

Administration method also matters. Miyawaki et al.^34^ showed that a single-syringe technique improved outcomes without increasing adverse events. Mohammadshahi et al.^35^ suggested external jugular access may enhance delivery, especially in low-resource settings. Patel et al.^36^ explored off-label digoxin use, though evidence remains limited.

Cost and side effects further support early mVM use. Wang et al.^25^ and Xiao et al.^26^ reported that early mVM success reduced drug use and healthcare costs. Xiao et al.^26^ proposed a hybrid protocol combining mVM with low-dose adenosine, achieving 86.1% overall success—a result that exceeds either intervention alone (mVM: 42%; adenosine: 75%) and should be interpreted as a distinct combined strategy rather than evidence of mVM efficacy in isolation.

In summary, while adenosine remains a key second-line agent, mVM provides a safer, cheaper, and more tolerable option—especially when paired with optimized protocols or used prehospital.

### Considerations in special populations and settings

Although the formal scope of this review is restricted to adult patients (≥18 years), brief contextual reference to special populations is warranted given their clinical relevance to emergency practice. It should be noted that the following observations fall outside the eligibility criteria of this review and are not part of the formal synthesis. Although mVM and pharmacologic agents like adenosine and verapamil are validated in general adult populations, certain clinical contexts require tailored approaches.

In children, vagal maneuvers are generally less effective. Abbasi et al.^31^ showed mVM outperformed other reflex-based techniques, though overall success was lower than in adults. Rayburn and Wagers^32^ reported a successful adaptation using a straw-assisted mVM in a pediatric case, highlighting its flexibility.

In pregnancy, non-pharmacological strategies are preferred to minimize fetal exposure. Patel et al.^36^ described effective use of carotid massage following failed drug treatment. Tednes et al.^4^ confirmed that adenosine remains safe when needed.

Resource-limited environments require adaptable interventions. Kassa et al.^37^ documented successful digoxin use in the absence of adenosine. Mohammadshahi et al.^35^ noted improved outcomes with central venous access, and Abbasi et al.^31^ recommended simplified delivery methods like single-syringe techniques.

Finally, prehospital feasibility—previously detailed in the subgroup analyses section—further reinforces mVM’s value as an intervention applicable across all care settings, including those with limited resources. In sum, mVM’s safety, simplicity, and adaptability make it a strong option across varied clinical contexts.

### Limitations of the study

This review has several limitations. First, although the eligibility criteria and search strategy were established prior to data extraction, the PROSPERO: CRD420251032213 was formally published in April 2025, after the literature search had been initiated in January 2025. This occurred as a result of an administrative oversight: the review protocol was designed and documented at the outset of the review, but formal submission to PROSPERO was not completed until April 2025. No amendments were made to the protocol following the search; the limitation is one of procedural transparency rather than methodological conduct.

Second, substantial clinical and methodological heterogeneity was identified across included studies. Sources of heterogeneity include: (1) variation in mVM protocol execution—differences in strain pressure targets, duration of strain phase, and leg elevation angle; (2) differences in outcome timing—cardioversion assessed at immediate, 1-min, 5-min, or unspecified intervals; (3) variability in the number of permitted attempts—ranging from a single attempt^22^ to three sequential attempts^12^^,^^25^; (4) heterogeneity in care settings—emergency department versus prehospital; and (5) differences in the definition of successful cardioversion. These factors precluded formal meta-analysis and must be considered when interpreting the direction and magnitude of reported effects. The moderate-to-high statistical heterogeneity reported in Lu et al.^15^ (I^2^ = 66%) further reflects this variability.

Third, the certainty of evidence was not formally assessed using GRADE or a “summary of findings” table. This decision was consistent with the narrative scope of the review and the degree of clinical and methodological heterogeneity that precluded pooled quantitative synthesis. However, we acknowledge that this limits the interpretability and transferability of our conclusions and that recommendations should be considered provisional rather than definitive pending future meta-analytic work with standardized protocols.

Fourth, special populations—such as children, older adults, and pregnant women—were underrepresented in the included studies, affecting generalizability.

Fifth, no formal assessment of reporting bias was conducted, as the absence of a meta-analysis precluded the use of funnel plots or equivalent tools. The possibility of publication bias cannot be excluded; the predominance of positive findings favoring mVM may partly reflect selective reporting, and this should be considered when interpreting the overall direction of evidence.

Finally, several included studies are single-center trials with modest sample sizes (*n* < 100), published in journals of varying impact. While the consistent direction of findings across diverse settings lends some credibility to the overall conclusions, the results from smaller studies should be interpreted with caution and considered hypothesis-generating rather than definitive. Larger, multicenter, adequately powered trials with standardized mVM protocols remain necessary to establish more robust estimates of effect.

### Implications for nursing practice and patient care

The mVM is a simple, safe, and effective intervention that trained nurses can autonomously apply, particularly in emergency and prehospital contexts. Its non-invasive nature and minimal equipment requirements make it especially valuable in rural and resource-limited settings. Nurses are ideally placed to lead education, training, and protocol development for mVM, thereby reducing pharmacological use, enhancing patient comfort, and improving arrhythmia management. Integrating mVM into routine practice supports a broader, evidence-based model of patient-centered cardiovascular care. Incorporating mVM protocols into nursing curricula and emergency training programmes may further enhance early arrhythmia management, reduce emergency department burden, and promote nurse-led innovation in cardiovascular care.

### Conclusions and recommendations for clinical practice

This systematic review supports the mVM as the most effective vagal technique for cardioversion in hemodynamically stable PSVT, with a consistently favorable safety profile. Direct head-to-head comparisons with pharmacological agents are limited; conclusions regarding its superiority over adenosine or CCBs should be interpreted as indirect inferences derived from separate evidence streams. Proper technique—including posture change and pressure generation—is essential for success.

Pharmacological treatments such as adenosine and CCBs remain effective second-line options, though their cost, side effects, and variable tolerability underscore the value of non-pharmacological strategies.

Contextual factors—age, pregnancy, and care setting—should guide treatment selection. The reproducibility and adaptability of mVM make it especially useful across clinical environments.

### Clinical recommendations

• First-line use: prioritize mVM in all stable PSVT cases.
• Training: ensure healthcare personnel are trained in correct mVM execution.
• Resource-aware implementation: promote mVM in settings with limited access to drugs or monitoring.
• Pharmacologic fallback: adenosine remains a reliable second-line therapy; optimize dose and route.
• Future research: standardize mVM protocols, explore adjunctive techniques, and conduct studies in special populations and low-resource settings.

In summary, mVM combines efficacy, safety, and versatility, positioning it as a cornerstone of non-invasive PSVT management. Its broader adoption—especially through nurse-led implementation—can improve outcomes and reduce healthcare burden. Furthermore, by empowering frontline staff to apply non-invasive strategies such as mVM, healthcare systems can improve patient comfort, reduce pharmacologic reliance, and foster more sustainable arrhythmia management.

## Resource availability

### Lead contact

Requests for further information should be directed to and will be fulfilled by the lead contact, Manuel Víctor López-González (manuelvictor@uma.es).

### Materials availability

This study did not generate new unique reagents or materials.

### Data and code availability

This systematic review did not generate new datasets or code. All data analyzed were extracted from the primary studies cited in the manuscript and are available in the original publications. Any additional information required to reanalyze the data reported in this paper is available from the lead contact upon request.

## Acknowledgments

The authors gratefully acknowledge the contributions of Jesús Rojano Bazuelo and Blanca Rubio Vergara, undergraduate nursing students at the University of Málaga. Their Final Degree Project provided the preliminary conceptual framework and early insights that supported the development of this systematic review. This study was supported by a program grant from the 10.13039/501100011011Junta de Andalucía, Research Group CTS-156. The authors also acknowledge the institutional support of the University of Málaga.

## Author contributions

All authors contributed substantially to the development of this systematic review. M.V.L.-G. acted as senior coordinator, overseeing the entire review process, verifying methodological decisions, synthesizing the final discussion, and leading the drafting and revision of the manuscript. He also coordinated team contributions and prepared the final version for submission. M.G.-G., a registered nurse currently employed as a lecturer in the Department of Nursing at the University of Málaga, and L.C.-F., a predoctoral researcher funded through the University of Málaga’s PIF program, critically appraised the included studies using CASPe and AMSTAR-II tools, ensured data extraction accuracy, and contributed clinical and methodological insight throughout the synthesis. C.M.-L., a predoctoral researcher under the FPU program of the Spanish Ministry of Science, contributed to data interpretation, refined the writing of the introduction and Methods sections, and verified the PRISMA flow diagram and eligibility tables. M.S.D.-M. served as external advisor, reviewing the structure, clarity, and scientific rigor of the manuscript, and provided expert input on autonomic and cardiovascular aspects relevant to clinical applicability. I.R., affiliated with the University of Lisbon, contributed to the conceptual design and methodological validation of the review protocol, particularly regarding its translational and international relevance. She also supported the discussion section and critically appraised neurophysiological evidence. All authors reviewed and approved the final version of the manuscript and agreed to be accountable for all aspects of the work. The authors affirm that the methods used in the data analyses are appropriate and have been correctly interpreted.

## Declaration of interests

The authors declare no competing interests.

## Declaration of generative AI and AI-assisted technologies in the manuscript preparation process

This manuscript benefited from English-language editing support provided by a large language model (ChatGPT, OpenAI), under the full supervision and responsibility of the authors.

## STAR★Methods

### Key resources table

**Table:** 

REAGENT or RESOURCE	SOURCE	IDENTIFIER
**Software and algorithms**		

Mendeley Reference Manager	Elsevier	https://www.mendeley.com

### Method details

#### Protocol and registration

This systematic review was conducted in accordance with the PRISMA 2020 guidance and was registered in PROSPERO: CRD420251032213. The review followed a two-phase protocol: (1) a comprehensive literature search and selection of eligible studies, and (2) a critical appraisal of methodological quality using validated tools. Only studies meeting minimum quality thresholds were included in the synthesis.

#### Eligibility criteria

Eligibility was defined a priori using the PICO model. Population: adults (≥18 years) with stable paroxysmal supraventricular tachycardia, irrespective of sex, comorbidities or care setting (emergency department or prehospital environment). Intervention: vagal maneuvers, including the standard and modified Valsalva techniques. Comparison: pharmacological cardioversion, primarily adenosine and, in some cases, calcium channel blockers. Outcome: return to sinus rhythm, tolerability, adverse effects and contraindications.Studies were eligible if published in English between December 2014 and December 2024, available as full-text peer-reviewed articles, designed as randomized controlled trials or systematic reviews/meta-analyses, comparative in nature, and conducted in adult human participants. Studies were excluded if they involved pediatric patients, pregnant women in the third trimester or hemodynamically unstable patients; focused on other arrhythmias; were case reports, editorials, narrative reviews or conference abstracts, or lacked an outcome comparison; or were of critically low methodological quality. One retrospective cohort study was included as an acknowledged deviation from these criteria, for the reasons set out in the STAR Methods.

#### Search and screening

Six databases were searched between January and April 2025: PubMed/MEDLINE, EMBASE, Cochrane Library, CINAHL, ProQuest Nursing & Allied Health Database and Scopus. Search strings combined controlled vocabulary (MeSH/Emtree) with free-text terms; the complete strategy for each database is given in Table 1. Results were limited to studies in adults published in English between December 2014 and December 2024. Duplicates were removed using Mendeley Reference Manager. Two reviewers screened titles, abstracts and full texts independently, and disagreements were resolved by discussion with a third reviewer. Additional sources were identified through backward citation tracking.

#### Critical appraisal

Methodological quality was appraised using tools selected according to study design. For randomized controlled trials and the retrospective cohort study, the CASPe checklist was used; this comprises 11 items, and studies were considered of acceptable quality if at least 7 items (≥64%) were met, with no critical domain unmet. For systematic reviews and meta-analyses, the AMSTAR-II instrument was applied, comprising 16 items; studies rated as “critically low” (more than one critical domain unmet) were excluded from the synthesis. Ratings are presented in Table 2 and were used to contextualize the weight given to individual findings, with results from studies with more unmet items discussed more cautiously and treated as exploratory rather than confirmatory.

#### Data extraction and synthesis

A standardized extraction form was completed by two independent reviewers, recording author, year and country; study design and sample size; patient characteristics and care setting; type of intervention and comparator; outcomes (cardioversion rate, recurrence, adverse effects); and key conclusions. Disagreements were resolved by a third reviewer. Owing to clinical and methodological heterogeneity, particularly in intervention protocols and outcome measures, meta-analysis was not appropriate and a narrative synthesis was performed, organized around four themes: effectiveness of standard versus modified Valsalva maneuvers; comparative efficacy of vagal versus pharmacological strategies; safety and tolerability of the interventions; and real-world applicability in emergency and prehospital settings. Only explicitly reported outcomes were analyzed; no imputation of missing data was performed, and no study authors were contacted.
